# Genome-Wide Association Study of COVID-19 Breakthrough Infections and Genetic Overlap with Other Diseases: A Study of the UK Biobank

**DOI:** 10.3390/ijms26136441

**Published:** 2025-07-04

**Authors:** Yaning Feng, Kenneth Chi-Yin Wong, Wai Kai Tsui, Ruoyu Zhang, Yong Xiang, Hon-Cheong So

**Affiliations:** 1 School of Medical Technology and Information Engineering, Zhejiang Chinese Medical University, Hangzhou 310053, China; fengyaningcuhk@link.cuhk.edu.hk; 2 School of Biomedical Sciences, The Chinese University of Hong Kong, Hong Kong SAR, Chinatwaikai@link.cuhk.edu.hk (W.K.T.); 1155128460@link.cuhk.edu.hk (R.Z.); xyong11@link.cuhk.edu.hk (Y.X.); 3 KIZ-CUHK Joint Laboratory of Bioresources and Molecular Research of Common Diseases, Kunming Institute of Zoology and The Chinese University of Hong Kong, Kunming 650204, China; 4 Department of Psychiatry, The Chinese University of Hong Kong, Hong Kong SAR, China; 5 CUHK Shenzhen Research Institute, Shenzhen 518172, China; 6 Margaret K.L. Cheung Research Centre for Management of Parkinsonism, The Chinese University of Hong Kong, Shatin, Hong Kong SAR, China; 7 Brain and Mind Institute, The Chinese University of Hong Kong, Hong Kong SAR, China; 8 Hong Kong Branch of the Chinese Academy of Sciences Center for Excellence in Animal Evolution and Genetics, The Chinese University of Hong Kong, Hong Kong SAR, China

**Keywords:** COIVD-19, breakthrough infection, genome-wide association study, genetic overlap

## Abstract

The coronavirus disease 2019 (COVID-19) pandemic has led to substantial health and financial burdens worldwide, and vaccines provide hope for reducing the burden of this pandemic. However, vaccinated people remain at risk for SARS-CoV-2 infection. Genome-wide association studies (GWASs) may identify potential genetic factors involved in the development of COVID-19 breakthrough infections (BIs); however, very few or no GWASs have been conducted for COVID-19 BI thus far. We conducted a GWAS and detailed bioinformatics analysis on COVID-19 BIs in a European population via the UK Biobank (UKBB). We conducted a series of analyses at different levels, including SNP-based, gene-based, pathway, and transcriptome-wide association analyses, to investigate genetic factors associated with COVID-19 BIs and hospitalized infections. The polygenic risk score (PRS) and Hoeffding’s test were performed to reveal the genetic relationships between BIs and other medical conditions. Two independent loci (LD-clumped at r^2^ = 0.01) reached genome-wide significance (*p* < 5 × 10^−8^), including rs36170929, which mapped to *LOC102725191*/*VWDE*, and rs28645263, which mapped to *RETREG1*. A pathway enrichment analysis highlighted pathways such as viral myocarditis, Rho-selective guanine exchange factor AKAP13 signaling, and lipid metabolism. The PRS analyses revealed significant genetic overlap between COVID-19 BIs and heart failure and between HbA1c and type 1 diabetes. Genetic dependence was also observed between COVID-19 BIs and asthma, lung abnormalities, schizophrenia, and type 1 diabetes on the basis of Hoeffding’s test. This GWAS revealed two significant loci that may be associated with COVID-19 BIs and a number of genes and pathways that may be involved in BIs. Genetic overlap with other diseases was identified. Further studies are warranted to replicate these findings and elucidate the mechanisms involved.

## 1. Introduction

COVID-19 has caused significant global health and economic impacts, with over 700 million cases and 7 million deaths reported as of January 2024 [1]. Vaccines remain the most effective strategy to reduce severe disease, mortality, and pandemic burden. They have also been shown to lower infection and transmission risks, especially before the emergence of the Omicron variants. However, vaccinated individuals can still experience breakthrough infections (BIs), raising critical questions about susceptibility factors despite vaccination.

BIs were relatively uncommon before Omicron, as vaccines provided strong protection against infection and severe disease. Those who developed BIs during this period may have unique genetic or clinical risk factors. Conversely, vaccination provided much weaker and rapidly waning protection against Omicron variants. For instance, effectiveness against Omicron infection dropped significantly after ~100 days [2] after vaccination; Lau et al. reported that vaccine effectiveness waned to 26% (95% CI: 7–41%) and 35% (95% CI: 10–71%) for three and four doses of BNT162b2 after 100 days. As such, this study focuses on BIs during the pre-Omicron period to identify genetic factors specifically linked to immune responses to vaccination, as the study of Omicron BIs may lead to the identification of variants linked to general infection susceptibility instead of vaccine responses.

Previous research [3,4] on BIs has largely focused on clinical risk factors, such as immune dysfunction or neutralizing antibody titers. However, genetic influences on BIs, particularly at the genome-wide level, remain underexplored. Understanding these genetic factors can provide insights into the mechanisms underlying poor vaccine responses, shed light on COVID-19 pathogenesis, and potentially guide drug repurposing.

**Figure 1:**
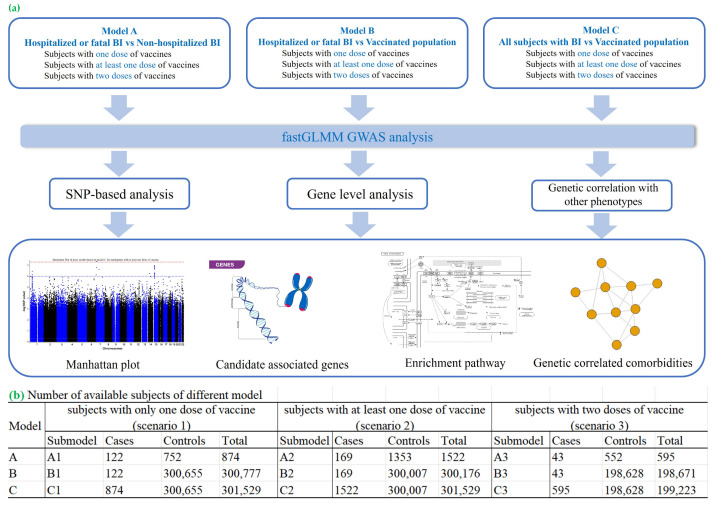
Workflow of our study and the number of available subjects of different models. (**a**) Overview of the analytical workflow, from participant selection to statistical modelling. (**b**) Sample sizes for each vaccination-dose model: participants who received exactly one dose, those with ≥1 dose, and those who completed a two-dose regimen.

Here, we conducted a genome-wide association study (GWAS) of COVID-19 BIs using UK Biobank data, focusing on pre-Omicron variants. To our knowledge, this is the first GWAS dedicated to investigating the genetic basis of BIs and severe infections during this period. Our study also compares severe and mild BI cases, complemented by extensive post-GWAS bioinformatics analyses. The workflow used in our study is shown in Figure 1a. We defined the study cohorts based on vaccination status and BI severity, including hospitalized and fatal cases. Our GWAS analyses identified genetic loci associated with BIs, revealing potential mechanisms underlying vaccine responses. Post-GWAS analyses, including gene-based analyses, pathway enrichment analyses, transcriptome-wide association studies (TWAS), and polygenic risk score (PRS) assessments, provided further insights. The PRS analyses uncovered links between BI-related genetic predispositions and other diseases, highlighting potential therapeutic opportunities.

This research offers a comprehensive view of the genetic architecture of COVID-19 BIs, presenting critical insights into immune responses to vaccination. These findings lay the groundwork for optimizing vaccines, understanding BI susceptibility, and developing targeted therapeutic interventions.

## 2. Results

### 2.1. Results from SNP-Based Analysis

#### 2.1.1. GWAS Results

A GWAS analysis across nine scenarios (Figure 1b) identified two loci significantly associated with COVID-19 BIs at the genome-wide level (*p* < 5 × 10^−8^) in cohort C (models C2 and C3, Figure 1b). These loci were rs36170929 on chromosome 7 (*p* = 4.39 × 10^−8^) and rs28645263 on chromosome 5 (*p* = 9.46 × 10^−9^). Manhattan plots for these GWASs are shown in Figures S1 and S2, with top SNPs listed in Table 1 and all SNPs with *p* < 1 × 10^−5^ in Tables S4–S12. Further details on genomic inflation factors (λ) and Quantile–Quantile (QQ) plots for all GWAS analyses are presented in Table S28 and Figure S5.

Sensitivity analyses were performed to assess the robustness of our findings. The results from the analyses using different r^2^ values for LD-clumping are summarized in Table S27. Additionally, sensitivity analyses incorporating vaccination date and type as covariates in Model C2 (Tables S25 and S26) yielded results highly consistent with the original analysis.

Additionally, we have performed post hoc power calculations for the two genome-wide significant SNPs (rs36170929 and rs28645263) using the genpwr R package (version 1.0.4). Post hoc power analyses indicated that our sample size (595 cases and 198,628 controls; based on Model C3) provided approximately 70% power to detect a genetic variant with a minor allele frequency of 0.416 and an odds ratio of 1.415 (beta = 0.347) at the genome-wide significance level of 5 × 10^−8^. Smaller effect sizes or lower-frequency variants would require larger sample sizes for adequate power. More detailed results are shown in Table S24.

#### 2.1.2. Significant SNPs Mapped to Genes

The rs36170929 locus maps to *LOC102725191*, an uncharacterized protein-coding gene. Based on the OpenTargets Genetics database, the top gene mapped to this SNP is *VWDE* (von Willebrand factor D and EGF domains; distance to this gene = 97.62 kb), as rs36170929 is an eQTL for *VWDE*. The rs28645263 locus maps to *RETREG1* (reticulophagy regulator 1). For the top 10 independent SNPs associated with COVID-19 BIs in Table 1, the most likely disease-associated genes corresponding to these SNPs were further prioritized by the overall V2G (variant-to-gene) score from OpenTargets Genetics (Table S13). Additional genes assigned via OpenTargets Genetics for SNPs with GWAS *p*-values < 1 × 10^−4^ are listed in Table S14. Region plots of rs36170929 and rs28645263 are shown in Figures S3 and S4, which display LD-clumped SNPs with these significant loci located within 1 Mb.

### 2.2. Results from Gene-Based Analysis

FastBAT analysis identified *BAGE* (*p* = 3.86 × 10^−8^, FDR = 9.51 × 10^−4^) as significantly associated with COVID-19 BIs. *BAGE2, BAGE3, BAGE4, BAGE5*, and *ARHGEF3* showed suggestive associations (FDR < 0.1, Table S15).

A pathway analysis (10,679 canonical pathways and GO gene sets) revealed significant associations for KEGG VIRAL MYOCARDITIS (FDR = 0.05), BIOCARTA AKAP13 PATHWAY (FDR = 0.06), KEGG TIGHT JUNCTION (FDR = 0.06), and REACTOME TRANSLATION (FDR = 0.06). Table 2 provides a summary, with detailed results in Tables S16 and S17. A GO gene set analysis highlighted significant associations for GOCC MUSCLE MYOSIN COMPLEX (FDR = 1.44 × 10^−5^) and GOCC MYOSIN COMPLEX (FDR = 6.41 × 10^−4^) in Model A (participants with at least one vaccine dose).

Using S-MulTiXcan, we investigated genetically regulated gene expression across 48 human tissues (Table S18). *AQP7P1* (FDR = 7.34 × 10^−3^), *PFN1P2* (FDR = 1.61 × 10^−2^), *AL590452.1*, and *LINC00842* (FDR < 0.05) were significantly associated with COVID-19 BIs. *RP11-314D7.3* showed moderate associations (FDR = 6.94 × 10^−2^). Additional results are in Table S19.

### 2.3. Results from Analysis of Genetic Overlap

The PRS analysis identified associations between COVID-19 BIs and other medical conditions (Table 3). For Model C2 (≥1 vaccine dose), the strongest association was with heart failure (FDR = 1.82 × 10^−3^), followed by HbA1c (FDR = 2.18 × 10^−2^) and type I diabetes (FDR = 1.22 × 10^−2^). Nominally significant associations (*p* < 0.05) were observed for obesity, BMI, dementia, asthma, COPD/asthma-related infections, and serum urate (Table S20).

Hoeffding’s test revealed significant genetic dependence between COVID-19 BIs and traits like asthma, abnormal lung imaging, type I diabetes, and schizophrenia (FDR < 0.05), while pulmonary embolism and cardiomyopathy showed FDR < 0.1. Nominally significant associations were identified for various cardiometabolic, neurological, and liver conditions (Table 4, Table S21).

PheWAS of the top 10 SNPs from models C2 and C3 revealed significant associations with lymphocyte and white blood cell (WBC) counts. Specifically, rs28645263 (*p* = 3.60 × 10^−4^) and rs9661909 (*p* = 2.64 × 10^−6^) were significantly associated with lymphocyte counts in PheWAS, with corresponding GWAS *p*-values of 9.46 × 10^−9^ and 1.56 × 10^−6^, respectively. Additionally, rs28645263 (*p* = 9 × 10^−4^) and rs4073656 (*p* = 1.23 × 10^−5^) were associated with white blood cell counts, with GWAS *p*-values of 9.46 × 10^−9^ and 9.89 × 10^−7^, respectively. Further details are provided in Tables S22 and S23.

## 3. Discussion

In this study, we conducted a GWAS to uncover the associated genetic factors of BIs using data from the UKBB. Furthermore, a series of post-GWAS analyses, including a gene-based analysis, a pathway enrichment analysis, a PRS analysis, etc., were performed to elucidate new insights into the genetic architecture of BIs. To our knowledge, this is the first GWAS to investigate the genetic basis of breakthrough COVID-19 infections (BIs) and severe infections, focusing on pre-Omicron variants, including a comparison of severe vs. mild BIs.

### 3.1. Interpretation of Findings

#### 3.1.1. Top Loci Identified via GWAS

We identified two loci, rs36170929 (*p* = 4.39 × 10^−8^) and rs28645263 (*p* = 9.46 × 10^−9^), significantly associated with COVID-19 BIs. These loci map to two genes: *LOC102725191* and *RETREG1* (*reticulophagy regulator 1*)*. RETREG1* is crucial in reticulophagy, a process that selectively eliminates portions of the endoplasmic reticulum (ER). Notably, a recent study [5] indicated that the ER-associated degradation (ERAD) regulator *ERLIN1* impedes the late-stage replication of the SARS-CoV-2 virus. *RETREG1*, along with *FNDC4*, also inhibits SARS-CoV-2 viral replication, suggesting that components of the ERAD pathway may serve as inhibitors of COVID-19 BIs.

While the overall sample size was large, the number of breakthrough infection cases was relatively small, which limited the statistical power in some models (~70%). This may have reduced our ability to detect weaker genetic signals and highlights the need for replication in larger cohorts or meta-analytic approaches. Nonetheless, key findings surpassed genome-wide significance and were supported by downstream bioinformatics analyses.

On the basis of OpenTargets, *VWDE* (von Willebrand factor D and EGF domains) was listed as the top gene mapped to rs3617092, considering that this SNP is an eQTL for *VWDE*. The von Willebrand factor (vWF) is a multimeric glycoprotein that is involved in inflammation and hemostasis. It has been reported that COVID-19 is associated with elevated levels of vWF antigen and activity, which may be linked to an increased risk of thrombosis in infected patients [6]. *VWDE* encodes a von Willebrand factor D and EGF domain-containing protein, which is implicated in extracellular matrix organization and cell adhesion. Given the important role of vascular integrity and endothelial function in COVID-19 pathophysiology, variation in VWDE may influence susceptibility to breakthrough infection by affecting vascular or immune responses. However, given the distance from the lead SNP and the lack of functional validation, this gene assignment remains tentative. Other genes in the region may also contribute to the observed association, and future studies incorporating chromatin interaction data and co-localization analysis will be important to clarify the causal gene(s) and mechanisms underlying this locus. For other loci, *KLF13* (*Kruppel-like factor 13*) shows low activity in moderate COVID-19 cases and higher activity in severe cases. Low *KLF13* expression correlates with reduced proinflammatory activity in macrophages, crucial for an efficient immune response. These results support the notion that *KLF13* is associated with COVID-19 severity [7].

#### 3.1.2. Gene-Based Results

Several *BAGE* family member genes, including *BAGE, BAGE2, BAGE3, BAGE4, and BAGE5*, were significantly associated with BIs according to the gene-based analysis. *BAGE* (B melanoma antigen) is a protein-coding gene. This gene encodes a tumor antigen recognized by autologous cytolytic lymphocytes (CTLs) [8]. There are currently no direct studies supporting the association between *BAGE* and COVID-19 or related diseases, and further studies are needed. In addition, *ARHGEF3* was observed to be associated with BIs. In another bioinformatics analysis [9] of differentially expressed gene targets in SARS-CoV-2 infection, *ARHGEF3* reached significance (*p*.adjust = 0.002415, Table 1 of reference [9]); however, further validation studies are needed.

### 3.2. Pathway and GO Enrichment Analysis

The most significant result in our pathway enrichment analysis was related to KEGG VIRAL MYOCARDITIS. Viral myocarditis is a cardiac disease associated with inflammation and injury of the myocardium. Myocarditis may be caused by direct cytopathic effects of the virus, a pathologic immune response to persistent virus, or autoimmunity triggered by the viral infection. Notably, viral myocarditis is associated with both COVID-19 infection and vaccination. According to a study in Israel, COVID-19 vaccination increased the 42-day risk of myocarditis by a factor of 3.24 (95% CI, 1.55–12.44) compared with unvaccinated individuals, with events mostly concentrated among young males [10]. Interestingly, viral myocarditis was identified as the top-ranked pathway, which may suggest that the genes involved in myocarditis are also associated with immunological responses to vaccination. The core subset of genes identified by GAUSS in this pathway could be a focus for further experimental studies, potentially providing new insights into associations between COVID-19 BIs and myocarditis [11]. However, while myocarditis is a rare but recognized adverse event of mRNA vaccination and COVID-19 infection, the involvement of myocarditis-related pathways may reflect shared immune or inflammatory mechanisms rather than a direct causal role in breakthrough infection risk itself. There remains a possibility of confounding due to post-vaccination myocarditis and/or myocarditis associated with COVID-19 infection. Further investigation is warranted to elucidate the underlying biological mechanisms.

Another pathway that also shows a suggestive association with BIs is the BIOCARTA AKAP13 PATHWAY (Rho-selective guanine exchange factor AKAP13 mediates stress fiber formation). A-kinase anchor protein 13 (*AKAP13*, also known as *AKAP-LBC*) is a group of structurally diverse proteins that bind to the regulatory subunit of protein kinase A (PKA) and confine the holoenzyme to discrete locations within the cell. A polymorphism near the *AKAP13* gene, associated with increased levels of *AKAP13* mRNA expression in the lung, was reported to be associated with an increased risk of developing idiopathic pulmonary fibrosis (IPF) [12]. Studies [13] have shown positive genetic correlations between IPF and COVID-19. In addition, *AKAP13* has been shown to regulate Toll-like receptor 2 (TLR2) signaling and play a role in innate immune responses downstream of TLRs [14].

Notably, lipid-related pathways, such as the WP LIPID METABOLISM PATHWAY and WP STEROL REGULATORY ELEMENT BINDING PROTEINS SREBP SIGNALLING, are also among the top pathways. Sterol regulatory element-binding proteins (SREBPs) are key regulators of lipid metabolism, including the synthesis of cholesterol. During viral infection, lipids play crucial roles in various processes, such as membrane fusion, replication, and endocytic and exocytic processes. Drugs that target lipid metabolism have also been suggested as drug targets [15].

In line with our findings that the PRS of diabetes-related traits are significantly associated with BIs, the “leptin-insulin signaling pathway overlap” was also a top-ranked pathway. Obesity is a well-known risk factor for severe COVID-19 infection, although the mechanism remains unclear. It has been postulated that leptin, which regulates both appetite and immunity [16], may contribute to the pathogenesis of COVID-19.

The interleukin-7 signaling pathway was also among the top pathways. Interleukin-7 (IL-7) is a cytokine crucial for T-cell development and homeostasis. IL-7 has been studied as a potential therapeutic for treating patients with severe COVID-19 with lymphopenia and lymphocyte exhaustion [17].

The differing findings across GWAS, gene-based, and pathway analyses reflect their methodological distinctions. GWAS detects individual SNPs with strong signals, gene-based analysis captures cumulative effects across gene regions, and pathway analysis identifies biologically related gene networks involved in disease susceptibility. These complementary approaches provide overlapping yet distinct insights, helping to explain why different but biologically related results may emerge, offering a more comprehensive view of the genetic architecture underlying COVID-19 breakthrough infections.

### 3.3. Polygenic Score Analysis and Genetic Overlap with Other Disorders

In the PRS association analysis, we observed a positive and significant genetic association between COVID-19 BIs and several traits, including heart failure and HbA1c (FDR < 0.05).

A recent study also revealed a positive genetic association between COVID-19 and heart failure [18]. Combined with our findings, these results provide evidence to support a partially shared genetic etiology between COVID-19 BIs and heart failure.

We also revealed a significant association between HbA1c and COVID-19 BIs. Interestingly, a related study [19] showed that poor glycemic control, assessed by mean HbA1c in the post-vaccination period, was associated with lower immune responses and an increased incidence of SARS-CoV-2 BIs in type 2 DM patients, consistent with our findings based on genetic data. Notably, we also observed significant genetic overlap between COVID-19 BIs and type I diabetes via both PRS and genetic dependence analyses with Hoeffding’s test. A recent review summarized the current studies on vaccine response and diabetes, with most studies reporting a lower antibody response in diabetic patients [20]; some studies reported that a higher BMI may also be associated with poorer immunogenicity. However, the high heterogeneity and modest sample sizes of many studies preclude a firm conclusion from being made.

A range of cardiometabolic traits were also nominally significant in our PRS or genetic dependence analyses, although they did not pass FDR correction. For example, obesity, BMI, diabetes mellitus (type I and II), and serum urate showed genetic overlap with BIs. As discussed above, several pathways related to lipid metabolism, leptin-insulin signaling overlap, etc., were among the top enriched pathways. Taken together, our results suggest that cardiometabolic traits share genetic bases with COVID-19 BIs. As such, it will be intriguing to study whether these cardiometabolic disorders are risk factors for or complications of COVID-19 BIs.

In the genetic dependence analysis with Hoeffding’s test, we observed several traits showing significant results passing FDR correction (FDR < 0.05), including asthma, abnormal findings on diagnostic imaging of the lung, schizophrenia, and type I diabetes. Given the possible genetic overlap between these traits and BIs, these traits may be linked to increased risks of BIs or present as sequelae post-infection. However, further studies are necessary to elucidate these relationships.

### 3.4. Other Related Studies

During the submission of this manuscript, we noted a recent related GWAS study on BIs [21]. However, the primary focus of our study is substantially different from the above work. We also wish to highlight that our findings were disseminated as publicly available preprints [22] months before the publication by Alcalde-Herraiz et al. [21].

Our study represents the first GWAS specifically investigating COVID-19 BIs during the pre-Omicron era. As explained earlier, given the low vaccine protection and rapidly waning immunity against Omicron variants, the study of BIs during the Omicron period likely results in the identification of variants linked to general infection susceptibility rather than vaccine-specific responses. In the above study by Alcalde-Herraiz et al. [21], they identified 74,662 subjects with BIs based on the UKBB, which represents ~24% of all eligible subjects (*N* = 315,323) for GWAS analyses. Such a high proportion of BIs supports the relatively low protection by vaccination during the Omicron period. As such, the identified loci may reflect overall tendencies to infection and may not be specific for vaccine responses.

Secondly, we uncovered novel loci (e.g., *RETREG1* and *VWDE*) and pathways, such as viral myocarditis and lipid metabolism, broadening the understanding of biological mechanisms underlying BIs. Thirdly, we have performed comprehensive post-GWAS analyses, including pathway enrichment, TWAS, polygenic risk analysis, and genetic dependence testing, providing a broader understanding of the biological mechanisms and genetic overlaps with other diseases. We revealed significant overlaps between BIs and cardiometabolic, respiratory, and neurological disorders, which may have important clinical implications. These comprehensive post-GWAS analyses and exploration of genetic overlap were not addressed in the Alcalde-Herraiz et al. [21] study. Taken together, despite related studies, our study presents unique findings and contributions to the field.

We also highlight a few other related genetic studies here, although their primary objective was on antibody responses post-vaccination, which differed from ours. Bian et al. [23] conducted a GWAS on the anti-spike IgG levels of UKBB participants who had not previously contracted SARS-CoV-2 infection and had received either the first or second dose of COVID-19 vaccines. Their work uncovered significant associations between IgG serostatus and human leukocyte antigen (HLA) class II alleles, demonstrating the protective role of the HLA-DRB1*13:02 allele. They also noted that the influence of HLA alleles on IgG responses was specific to cell types. Similarly, Mentzer et al. [24] performed GWAS of antibody (anti-receptor-binding domain (RBD)) responses 28 days after ChAdOx1 nCoV-19 vaccination. Seroconversion response was also studied by Alcalde-Herraiz et al. [21] in their GWAS analyses.

While these studies focused on antibody responses after vaccination, our research takes a distinct approach by investigating breakthrough infections (BIs) across different vaccine doses and their severity. Although antibody responses have been linked to risks of BIs, they do not fully explain the risks of such infections. For example, Aldridge et al. [25] reported that each unit increase in (log-transformed) anti-S levels post-vaccination was associated with a reduced hazard ratio (HR) of 0.85. Given the modest effect size, there are likely other factors contributing to the heterogeneity of risks of BIs. Importantly, we also identified a genetic overlap between COVID-19 BIs and a wide variety of other diseases, an aspect not covered in previous studies.

### 3.5. Strengths and Limitations

Firstly, to the best of our knowledge, this is the first GWAS to investigate the genetic basis of breakthrough COVID-19 infections (BIs) and severe infections (focusing on pre-Omicron variants), including a comparison of severe vs. mild BIs. Secondly, we conducted a comprehensive series of post-GWAS analyses to provide insights into the biological basis of COVID-19 BIs. These include standard SNP-based tests as well as gene-based (fastBAT, S-MulTiXcan) and pathway-based (GAUSS) analyses, which may help bridge the gap between the significant SNPs detected and their corresponding biological mechanisms. Finally, we explored the genetic associations between COVID-19 BIs and related disorders through PRS and other analyses.

Our study has a few limitations. Firstly, although the total sample size in our study was large, the number of cases was relatively limited because of the relatively short follow-up duration (a maximum of 253 days between vaccination and infection). However, studies have shown that the effectiveness of vaccines in preventing infection wanes over time [26]. This challenge makes it more difficult to capture specific genetic factors underlying the vaccine response as the follow-up length increases. We aimed to balance follow-up length and vaccine effectiveness to determine the genetics of BIs. Additionally, the UK Biobank population may not fully represent the entire UK population, as participants tend to be healthier and have higher socioeconomic status than non-participants do. Furthermore, our study is based on European samples, and the generalizability of these genetic findings to other populations remains uncertain. Further studies in other populations are warranted. Extreme case-control imbalance may reduce the power to detect modest associations, though fastGWA-GLMM helps mitigate bias via a generalized linear mixed model. Replication in independent datasets remains necessary. Different vaccine types may trigger immune responses through distinct pathways, potentially affecting genetic associations with breakthrough infections. Larger studies stratified by vaccine type are needed to validate these findings and investigate vaccine-specific genetic interactions.

In summary, we conducted a GWAS for breakthrough infections with the SARS-CoV-2 virus in a European population using UK Biobank. A series of post-GWAS analyses were performed, including a gene-based analysis, a pathway enrichment analysis, a PRS association, and others. We discovered two novel genetic loci and revealed corresponding genes and pathways that may underlie COVID-19 BIs. We believe that this work provides an important foundation for future studies attempting to elucidate the biological and genetic basis of COVID-19 breakthrough infections.

## 4. Materials and Methods

### 4.1. Data Source

The individual-level data were extracted from the UK Biobank (UKBB), a large-scale prospective cohort with ~500,000 participants aged 50–89 years. This study was conducted under UKBB project number 28732 [27].

### 4.2. COVID-19 Infection Status

COVID-19 infection data were obtained from the UKBB data portal, last updated on 21 July 2021. Infection status was determined through test results, ICD-code U071 from hospital inpatient or mortality records, or the code “Y2a3b” in TPP General Practice clinical records. COVID-19 cases in this study were defined as laboratory-confirmed infections.

### 4.3. Vaccination Status

Vaccination records were sourced from TPP and EMIS GP clinical systems. Most participants received either the BioNTech BNT162b2 or Oxford-AstraZeneca ChAdOx1 nCoV-19 vaccine. The participants were categorized based on vaccination status: one dose, at least one dose, and two doses. The median follow-up for vaccinated individuals was 54 days.

### 4.4. Inclusion and Exclusion Criteria

The study included vaccinated individuals (N = 393,544) without prior COVID-19 infections. Those with imputed genotype data labeled as European ancestry (UKBB data field 22006) were included.

### 4.5. Phenotype Definition

A breakthrough infection (BI) was defined as a COVID-19 infection occurring 14 days post-vaccination. Cohorts A, B, and C were established, as shown in Table S1. Cohort A compared hospitalized or fatal BIs with non-hospitalized BIs. Cohort B compared hospitalized or fatal BIs to individuals without COVID-19 BIs. Cohort C compared all BI cases to individuals without BIs. The number of cases and controls in each model is shown in Figure 1b. For example, in Model C2—where a BI is defined as an infection occurring after at least one dose of vaccine in cohort C—we identified 1522 cases and 300,007 controls.

### 4.6. Genotyping and Quality Control (QC)

Genotyping was performed using the Applied Biosystems UK BiLEVE Axiom Array (Affymetrix, now part of Thermo Fisher Scientific, Waltham, MA, USA), and genotype data were imputed and aligned to the GRCh37 reference genome [28]. QC excluded variants with minor allele frequencies < 1%, missingness > 10%, and Hardy–Weinberg equilibrium *p*-values < 1 × 10^−10^. After QC, 485,623 common variants with MAFs > 0.01 and 488,371 individuals remained for the analysis. Imputed variants meeting the standard criteria were retained for GWAS (Table S2).

### 4.7. Genome-Wide Association Study (GWAS)

GWAS was conducted using fastGWA-GLMM [29] to test for associations between imputed SNP dosages and BI phenotypes in each cohort. This tool calculates a sparse genomic relationship matrix to evaluate the pedigree relatedness among individuals, thereby controlling for family structure without the need to exclude related individuals. In addition, fastGWA-GLMM can handle imbalanced data (e.g., when cases are rare compared with the controls). We fitted age, sex, age × age, age × sex, and the top 10 genetic principal components provided by UKBB (data field 22009) as covariates. Also, vaccination date (measured as days since the start of the vaccination campaign) and vaccine type were added as covariates as part of the sensitivity analysis based on Model C2. For those with missing vaccine type data, we imputed the variable using a multinomial logistic regression approach using the mice R package (version 3.17.0).

### 4.8. SNP-Based Analysis

We conducted SNP-level GWAS using the fastGWA-GLMM framework, as described above, to identify common genetic variants associated with COVID-19 BIs. Imputed SNP dosages passing QC filters (details are shown in Section 4.6) were included. Genome-wide statistical significance was defined using the conventional threshold of *p* < 5 × 10^−8^. To identify independent loci, LD-clumping was further performed via PLINK 1.9 (r^2^ = 0.5, distance = 250 kb) to identify the independent loci. European samples in the Phase 3 1000-Genomes Project were used as the LD reference (GRCh37) [30]. SNP-to-gene mapping was performed via the Bioconductor package “biomaRt” (version 2.48.2) in R-4.0.3. A post hoc power analysis using the genpwr R package (version 1.0.4) was conducted further. Also, the OpenTargets Genetics portal [31] was employed to prioritize the most relevant genes for each variant as a supplementary analysis.

### 4.9. Gene Set and Pathway Analyses

Gene-based tests were conducted with fastBAT, using LD reference data from 1000 genomes [32]. Pathway and gene ontology (GO) enrichment analyses were performed with GAUSS [33]; we also identified core subsets of genes contributing to significant associations. Multiple testing was controlled using the Benjamini–Hochberg FDR method with thresholds of 0.05 (significant) and <0.1 (suggestive association).

### 4.10. Transcriptome-Wide Association Studies (TWASs)

TWAS provides a novel approach for gene—trait association studies. TWAS utilizes known genetic variants (eQTLs) associated with transcript abundance to infer gene expression from GWAS data, thereby exploring associations between genetically regulated expression and complex traits. TWAS was conducted for 48 tissues using S-PrediXcan [34] with GTEx v8 data. We also performed a meta-TWAS using S-MulTiXcan, integrating results across tissues to improve the statistical power [35].

### 4.11. Phenome-Wide Association Studies (PheWASs)

PheWASs investigated associations between identified SNPs and a broad range of phenotypes using summary statistics from UKBB, FinnGen, and GWAS Catalog via OpenTargets Genetics [31].

### 4.12. Polygenic Risk Score (PRS) Analysis

PRS analyses [36] explored the genetic overlap of COVID-19 BIs with other conditions (e.g., asthma, cardiovascular diseases, and diabetes) using FinnGen summary statistics to avoid overlap with UKBB samples. SNP selection thresholds ranged from 5 × 10^−8^ to 0.01, with LD-clumping performed at r^2^ = 0.05 within a 250 kb distance.

### 4.13. Genetic Dependence Analysis

We employed Hoeffding’s test to evaluate the genetic dependence between COVID-19 breakthrough infections (BIs) and other diseases. This nonparametric method examines the marginal and joint distributions of two variables, avoiding parametric assumptions, and is particularly suited for small or moderate sample sizes. Clumping was performed via PLINK (distance threshold of 10,000 kb; r^2^ = 0.2). The genetic dependence was tested across various conditions, including respiratory, cardiovascular, endocrine, and neurological disorders (see Table S3). The R package “independence” was used following procedures described in prior studies [37].

## Figures and Tables

**Table 1:** Top 10 SNP-based results in Model C.

Models	SNP	Chr.	Location (bp)	Effect Allele	Non-Effect Allele	Frequency of Effect Allele	BETA	SE	*p*	N	INFO	Gene Symbol	Gene Name	Total no. of SNPs from LD-Clumping	S0001	Top Gene Prioritized by OpenTargets
Participants with ***two doses of vaccine***	rs28645263	5	16612885	C	T	0.416	0.347	0.06	***9.46 × 10^−9^***	199,223	0.964	*RETREG1*	reticulophagy regulator 1	3	3	*RETREG1*
	rs4073656	2	48981646	G	A	0.502	−0.288	0.059	9.89 × 10^−7^	199,223	0.988	*LHCGR*	luteinizing hormone/choriogonadotropin receptor	5	3	*STON1-GTF2A1L*
	rs9661909	1	206714818	T	C	0.506	−0.282	0.059	1.56 × 10^−6^	199,223	0.985	*RASSF5*	Ras association domain family member 5	11	6	*RASSF5*
	rs72718228	14	69475527	T	C	0.09	0.493	0.105	2.49 × 10^−6^	199,223	1			5	4	*ACTN1*
	rs4991425	10	123485856	T	C	0.363	−0.288	0.061	2.62 × 10^−6^	199,223	0.983			10	8	*FGFR2*
	rs111692702	19	15651802	A	G	0.009	1.729	0.371	3.21 × 10^−6^	199,223	0.97	*CYP4F22*	cytochrome P450 family 4 subfamily F member 22	3	3	*CYP4F22*
	rs28718712	17	29882071	T	G	0.671	−0.287	0.062	3.60 × 10^−6^	199,223	1			32	4	*RAB11FIP4*
	rs4687124	3	189840935	G	A	0.232	0.319	0.07	4.86 × 10^−6^	199,223	0.998			22	22	*P3H2*
	rs2874139	4	169751502	C	G	0.68	−0.288	0.063	5.49 × 10^−6^	199,223	0.979	*PALLD*	palladin, cytoskeletal associated protein	43	15	*PALLD*
	rs12466174	2	184802609	T	G	0.122	0.417	0.092	5.81 × 10^−6^	199,223	0.969			8	7	*NA*
Participants with ***at least one dose of vaccine***	rs36170929	7	12541187	G	A	0.64	0.21	0.038	***4.39 × 10^−8^***	301529	0.984254			11	5	*VWDE*
	rs56150535	15	31647722	T	C	0.359	0.203	0.038	1.09 × 10^−7^	301,529	0.996787	*KLF13*	Kruppel like factor 13	33	20	*KLF13*
	rs181987785	1	34977912	G	A	0.005	1.449	0.284	3.48 × 10^−7^	301,529	0.984316			30	30	*GJB5*
	rs187268954	3	116529463	C	T	0.004	1.736	0.358	1.22 × 10^−6^	301,529	0.90264			4	3	*LSAMP*
	rs7590599	2	108915136	C	T	0.604	0.182	0.038	1.26 × 10^−6^	301,529	0.989482	*SULT1C2*	sulfotransferase family 1C member 2	8	5	*SULT1C2*
	rs3737328	13	110866065	T	C	0.246	0.198	0.042	3.05 × 10^−6^	301,529	1	*COL4A1*	collagen type IV alpha 1 chain	4	4	*COL4A1*
	rs142193221	22	21166165	A	G	0.006	1.274	0.274	3.31 × 10^−6^	301,529	0.929992	*PI4KA*	phosphatidylinositol 4-kinase alpha	6	6	*PI4KA*
	rs56070971	1	35025879	T	C	0.006	1.275	0.276	3.86 × 10^−6^	301,529	0.968667			29	29	*GJB5*
	rs72664942	4	85808904	G	A	0.007	1.174	0.259	5.75 × 10^−6^	301,529	0.938787	*WDFY3*	WD repeat and FYVE domain containing 3	2	2	*WDFY3*
	rs79158353	10	78798475	A	T	0.082	−0.304	0.067	6.48 × 10^−6^	301,529	0.995184	*KCNMA1*	potassium calcium-activated channel subfamily M alpha 1	23	13	*KCNMA1*

(1) S0001, number of clumped SNPs (SNPs in LD) with *p* < 1 × 10^−3^; only SNPs with S0001 ≥ 2 are shown. (2) LD-clumping settings: r^2^ = 0.5, distance = 250 kb. (3) Bold and italicized *p*-values indicate genome-wide significance (*p* < 5 × 10^−8^).

**Table 2:** Top 15 pathway enrichment results (GAUSS) for genes identified through gene-based analysis (fastBAT).

GeneSet	Length_GS	*p*-Value	Excluded	*p*_Adjust_BH	Model
KEGG_VIRAL_MYOCARDITIS	41	9.05 × 10^−6^	22	5.69 × 10^−2^	A1
BIOCARTA_AKAP13_PATHWAY	21	9.91 × 10^−6^	1	6.23 × 10^−2^	B2
KEGG_TIGHT_JUNCTION	73	1.38 × 10^−5^	11	6.29 × 10^−2^	A2
REACTOME_TRANSLATION	295	2.00 × 10^−5^	76	6.29 × 10^−2^	B2
REACTOME_MITOCHONDRIAL_TRANSLATION	96	1.10 × 10^−4^	4	1.73 × 10^−1^	A2
REACTOME_PASSIVE_TRANSPORT_BY_AQUAPORINS	13	8.00 × 10^−5^	0	5.03 × 10^−1^	C3
MYLLYKANGAS_AMPLIFICATION_HOT_SPOT_29	33	1.60 × 10^−4^	0	5.35 × 10^−1^	C1
YAMASHITA_LIVER_CANCER_WITH_EPCAM_DN	53	1.70 × 10^−4^	0	5.35 × 10^−1^	C1
APRELIKOVA_BRCA1_TARGETS	48	2.00 × 10^−4^	8	7.17 × 10^−1^	C2
WP_LEPTIN_INSULIN_OVERLAP	30	2.50 × 10^−4^	1	7.17 × 10^−1^	C2
REACTOME_INTERLEUKIN_7_SIGNALING	9	3.90 × 10^−4^	13	7.17 × 10^−1^	C2
WP_LIPID_METABOLISM_PATHWAY	23	3.40 × 10^−4^	0	7.76 × 10^−1^	B1
WP_STEROL_REGULATORY_ELEMENTBINDING_PROTEINS_SREBP_SIGNALLING	8	3.70 × 10^−4^	4	7.76 × 10^−1^	B1
REACTOME_PI3K_AKT_ACTIVATION	9	2.90 × 10^−4^	1	7.97 × 10^−1^	C3
WP_STRIATED_MUSCLE_CONTRACTION_PATHWAY	11	3.00 × 10^−4^	2	8.39 × 10^−1^	A1

**Table 3:** Polygenic association testing of BIs (Model C2, general BIs vs. population) with related traits via summary statistics (*p* < 0.05 are shown).

Body System	Exposure	pval_PRS	*p*_adjust_BH	Coefficient	r2	nsnps	exposure_p_filter	clump_r2
cardiovascular system	Heart Failure	***1.33 × 10^−4^***	***1.82 × 10^−3^***	0.030588	4.84 × 10^−5^	41,900	0.05	0.05
endocrine system	Type 1 diabetes, strict (exclude type 2)	***1.00 × 10^−3^***	***1.22 × 10^−2^***	0.028586	3.59 × 10^−5^	131	5.00 × 10^−8^	0.05
endocrine system	Glycaemic_HbA1c	***1.96 × 10^−3^***	***2.18 × 10^−2^***	0.704835	3.18 × 10^−5^	250	1.00 × 10^−4^	0.05
endocrine system	Diabetes mellitus (type 1 and 2)	***1.61 × 10^−2^***	1.30 × 10^−1^	0.097242	1.92 × 10^−5^	128	5.00 × 10^−8^	0.05
endocrine system	Obesity	***3.63 × 10^−2^***	2.37 × 10^−1^	0.004535	1.45 × 10^−5^	56,424	0.05	0.05
endocrine system	BMI	***1.49 × 10^−2^***	1.32 × 10^−1^	0.17485	1.97 × 10^−5^	1365	1.00 × 10^−7^	0.05
immune system	Human immunodeficiency virus disease	***3.71 × 10^−2^***	2.41 × 10^−1^	0.042006	1.44 × 10^−5^	17	1.00 × 10^−5^	0.05
nervous system	Dementia	***2.95 × 10^−2^***	2.00 × 10^−1^	0.003864	1.57 × 10^−5^	78,932	0.1	0.05
respiratory system	COPD/asthma-related infections	***9.15 × 10^−3^***	***8.58 × 10^−2^***	0.01321	2.25 × 10^−5^	54,680	0.05	0.05
respiratory system	Asthma	***2.09 × 10^−2^***	1.62 × 10^−1^	−0.009499	1.77 × 10^−5^	20,426	0.01	0.05
respiratory system	Smoking Cessation	***4.00 × 10^−2^***	2.46 × 10^−1^	0.15793	1.40 × 10^−5^	2871	0.001	0.05
renal system	Diabetic kidney disease in type 1 DM	***9.75 × 10^−3^***	***9.00 × 10^−2^***	−0.015166	2.22 × 10^−5^	1449	0.001	0.05
renal system	Serum urate	***1.16 × 10^−2^***	1.04 × 10^−1^	1.205126	2.11 × 10^−5^	33	0.05	0.05

(1) clump_r2 = 0.05. (2) More details about the information for each exposure are listed in Table S3. (2) All of the outcomes in this table are Model C2, defined in Figure 1b. (3) Bolded and italicized values in the *p*val_PRS column indicate nominal statistical significance (*p* < 0.05). Bolded and italicized values in the *p*_adjust_BH column indicate statistical significance after Benjamini–Hochberg FDR correction for multiple testing (FDR adjusted *p* < 0.1).

**Table 4:** Hoeffding’s independence test of BIs with related traits via summary statistics (*p* < 0.05 are shown).

Exposure	Outcome	pthres	n	Dn	Scaled	*p*. Value	*p*.adj_pthres&traitB_Separate
Respiratory							
Abnormal findings on diagnostic imaging of lung	A2	0.1	102,776	1.29 × 10^−6^	4.76	**2.05 × 10^−4^**	**8.00 × 10^−3^**
Abnormal findings on diagnostic imaging of lung	B2	0.1	102,787	7.19 × 10^−7^	2.66	**4.47 × 10^−3^**	1.74 × 10^−1^
Asthma (only as main diagnosis)	A2	0.5	372,099	1.94 × 10^−7^	2.6	**4.88 × 10^−3^**	1.43 × 10^−1^
Asthma (only as main diagnosis)	B2	0.5	372,141	1.81 × 10^−7^	2.42	**6.41 × 10^−3^**	**8.33 × 10^−2^**
Asthma (only as main diagnosis)	C2	0.05	68,429	1.55 × 10^−6^	3.82	**8.08 × 10^−4^**	**2.69 × 10^−2^**
Asthma, hospital admissions, main diagnosis only	A2	0.5	371,828	1.63 × 10^−7^	2.19	**9.11 × 10^−3^**	1.43 × 10^−1^
COPD/asthma-related infections	B2	1.00 × 10^−5^	44	8.24 × 10^−4^	1.28	**3.77 × 10^−2^**	2.56 × 10^−1^
COPD/asthma-related pneumonia or pneumonia-derived septicaemia	A2	0.01	15,042	2.35 × 10^−6^	1.27	**3.81 × 10^−2^**	2.97 × 10^−1^
Interstitial lung disease	A2	0.3	248,253	2.02 × 10^−7^	1.81	**1.63 × 10^−2^**	3.08 × 10^−1^
Interstitial lung disease endpoints	C2	0.2	190,993	1.70 × 10^−7^	1.17	**4.49 × 10^−2^**	6.65 × 10^−1^
Obesity-related asthma	A2	0.01	15,347	3.12 × 10^−6^	1.73	**1.85 × 10^−2^**	2.41 × 10^−1^
Obesity-related asthma	B2	0.01	15,350	2.12 × 10^−6^	1.17	**4.48 × 10^−2^**	5.82 × 10^−1^
Pulmonary embolism	B2	0.05	58,577	1.46 × 10^−6^	3.07	**2.43 × 10^−3^**	**6.17 × 10^−2^**
Tuberculosis	A2	0.01	13,123	3.08 × 10^−6^	1.45	**2.84 × 10^−2^**	2.77 × 10^−1^
**Cardiovascular**							
Cardiomyopathy	C2	0.1	103,175	9.18 × 10^−7^	3.41	**1.48 × 10^−3^**	**5.75 × 10^−2^**
Cardiomyopathy (excluding other)	B2	0.5	363,183	1.87 × 10^−7^	2.44	**6.18 × 10^−3^**	**8.33 × 10^−2^**
Cardiomyopathy (no controls excluded)	A2	0.01	14,204	4.44 × 10^−6^	2.27	**8.07 × 10^−3^**	1.57 × 10^−1^
**Endocrine**							
Diabetes mellitus (type 1 and 2)	A2	0.3	275,918	1.17 × 10^−7^	1.16	**4.52 × 10^−2^**	3.52 × 10^−1^
Diabetes mellitus (type 1 and 2)	C2	0.1	129,409	4.66 × 10^−7^	2.17	**9.33 × 10^−3^**	1.82 × 10^−1^
Obesity	B2	0.4	319,934	1.34 × 10^−7^	1.54	**2.49 × 10^−2^**	3.95 × 10^−1^
Type 1 diabetes, strict definition	A2	1.00 × 10^−4^	728	9.35 × 10^−5^	2.45	**6.16 × 10^−3^**	1.20 × 10^−1^
Type 1 diabetes, wide definition	B2	0.2	179,971	6.59 × 10^−7^	4.27	**4.21 × 10^−4^**	**1.64 × 10^−2^**
Type 1 diabetes, wide definition	C2	0.05	56,637	6.89 × 10^−7^	1.41	**3.07 × 10^−2^**	3.99 × 10^−1^
**Neurological**							
Schizophrenia or delusion	C2	1.00 × 10^−5^	35	2.00 × 10^−3^	2.44	**6.19 × 10^−3^**	2.41 × 10^−1^
Schizophrenia or delusion (more controls excluded)	A2	0.01	15,032	6.43 × 10^−6^	3.48	**1.33 × 10^−3^**	**5.20 × 10^−2^**
Schizophrenia, schizotypal and delusional disorders	B2	1.00 × 10^−5^	43	3.09 × 10^−3^	4.68	**2.33 × 10^−4^**	**9.09 × 10^−3^**
Any dementia	B2	1.00 × 10^−5^	109	4.62 × 10^−4^	1.8	**1.66 × 10^−2^**	2.56 × 10^−1^
Any dementia (more controls excluded)	A2	0.001	1858	2.18 × 10^−5^	1.45	**2.84 × 10^−2^**	2.77 × 10^−1^
**Liver**							
Alcoholic liver disease	A2	0.001	1690	3.69 × 10^−5^	2.25	**8.35 × 10^−3^**	2.77 × 10^−1^
Cirrhosis, broad definition	A2	1.00 × 10^−4^	202	3.92 × 10^−4^	2.84	**3.43 × 10^−3^**	1.20 × 10^−1^
Cirrhosis, broad definition	C2	0.3	248,811	1.36 × 10^−7^	1.22	**4.12 × 10^−2^**	6.57 × 10^−1^
Nonalcoholic fatty liver disease	B2	0.2	178,801	1.82 × 10^−7^	1.17	**4.45 × 10^−2^**	4.34 × 10^−1^

(1) More details about the information for each exposure are listed in Table S3. (2) Scaled statistic: the test statistic rescaled for a standard null distribution (please refer to the R package “independence” for details). FDR-adjusted *p*-values(*p*.adj_pthres&traitB_Separate) < 0.1 are in bold. FDR adjustment was performed with stratification by trait B. (3) The r^2^ threshold for LD-clumping is 0.2. (4) The definitions of the outcomes are listed in Figure 1b.

## Data Availability

The original contributions presented in this study are included in the article/Supplementary Materials. Further inquiries can be directed to the corresponding author.

## Supplementary Materials

The following supporting information can be downloaded at https://www.mdpi.com/article/10.3390/ijms26136441/s1.
