# Supplementary material for: Genome-Wide Association Study of COVID-19 Breakthrough Infections and Genetic Overlap with Other Diseases: A Study of the UK Biobank
Source: Int J Mol Sci. 2025 Jul 4;26(13):6441. doi: 10.3390/ijms26136441 (PMC12249495; doi:10.3390/ijms26136441)
Supplement: Supplementary file 1 [file ijms-26-06441-s001.zip › Supplementary_files.pdf]

Supplementary Files

All supplementary files are available at:
https://drive.google.com/drive/folders/1GXVRuE9l1axRXUL63D61Cyagr9UvsKCv?usp=sharing

Supplementary Tables

Table S1 Definitions of models for covid-19 breakthrough infections
Table S2 Summary of the number of variants in each scenario
Table S3 Summary of base GWAS data for polygenic risk score analysis
Table S4 Results of SNP-based analysis (p<1e-3) based on Model A for participants with only one dose of vaccine
1) S0001, number of clumped SNPs (SNPs in LD) with p<1e-3; only SNPs with S0001 >= 2 are shown.
2) LD clumping settings: r2=0.5, distance = 250 kb
Table S5 Results of SNP-based analysis (p<1e-3) based on Model B for participants with only one dose of vaccine
Table S6 Results of SNP-based analysis (p<1e-3) based on Model C for participants with only one dose of vaccine
Table S7 Results of SNP-based analysis (p<1e-3) based on Model A for participants with at least one dose of vaccine
Table S8 Results of SNP-based analysis (p<1e-3) based on Model B for participants with at least one dose of vaccine
Table S9 Results of SNP-based analysis (p<1e-3) based on Model C for participants with at least one dose of vaccine
Table S10 Results of SNP-based analysis (p<1e-3) based on Model A for participants receiving two doses of vaccine
Table S11 Results of SNP-based analysis (p<1e-3) based on Model B for participants receiving two doses of vaccine
Table S12 Results of SNP-based analysis (p<1e-3) based on Model C for participants receiving two doses of vaccine
Table S13 Results of assigned genes from OpenTargetGenetics for the top 10 SNPs in Model C (participants with two doses of vaccine and at least one dose of vaccine)
Table S14 Results of assigned genes from OpenTargetGenetics for SNPs with p values of GWASs less than 1e-4
Table S15 Top 15 results of gene-based analysis based on all the models used in our study
Table S16 Top 20 results of pathway enrichment results (GAUSS) for genes from gene-based analysis (fastBAT)
Table S17 Top 20 results of the GO enrichment results (GAUSS) for genes from the gene-based analysis (fastBAT)
Table S18 List of tissues included in our TWAS analysis
Table S19 Top 20 results of TWAS analysis with S-multiXcan on the basis of all the scenarios in our study

Table S20 Polygenic association testing of BI (participants with at least one dose of vaccine) with related traits FinnGen via summary statistics
Table S21 Hoeffding's D Independence test of BI (participants with at least one dose of vaccine) with related traits via summary statistics
Table S22 Results of PheWAS analysis for the top 10 SNPs in Model C (participants with two doses of vaccine, at least one dose of vaccine)
1) S0001, number of clumped SNPs (SNPs in LD) with p<1e-3; only SNPs with S0001 >= 2 are shown.
2) LD clumping settings: r2=0.5, distance = 250 kb
3) Summary statistics for PheWAS include UK Biobank, FinnGen, and/or GWAS Catalog summary statistics
Table S23 Results of PheWAS analysis for SNPs with p values of GWASs less than 1e-5
Table S24 Post-hoc power calculations for the two genome-wide significant SNPs in our study
Table S25 GWAS results with vs. without vaccination date adjustment in model C2
Table S26 GWAS results with vs. without vaccination type adjustment in model C2
Table S27 Top 10 SNPs associations from model C under varying LD clumping thresholds (r^2) among participants with at least one vaccine dose
Table S28 Genomic inflation factors (λ) for GWAS analyses across study scenarios

Supplementary Figures

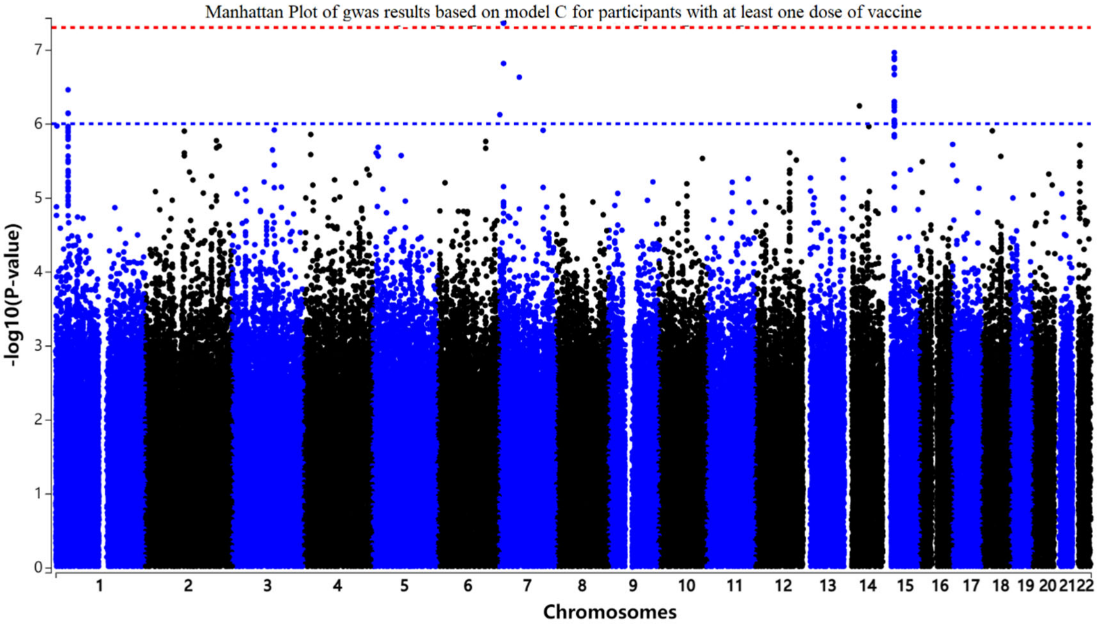

Fig. S1 Manhattan plot of GWAS results based on Model C for participants who received at least one dose of vaccine
Note: ‘Model C for participants with at least one dose of vaccine’ is C2 defined in Figure 1b.

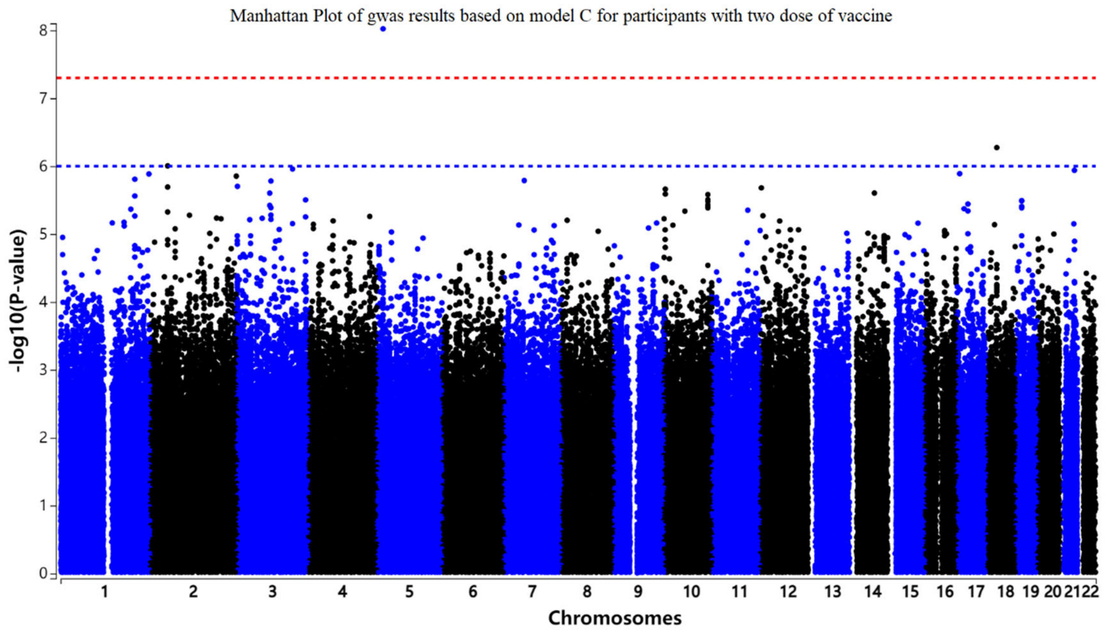

Fig. S2 Manhattan plot of GWAS results based on Model C for participants receiving two doses of vaccine

Note: 'Model C for participants with two doses of vaccine' is C3 defined in Figure 1b.

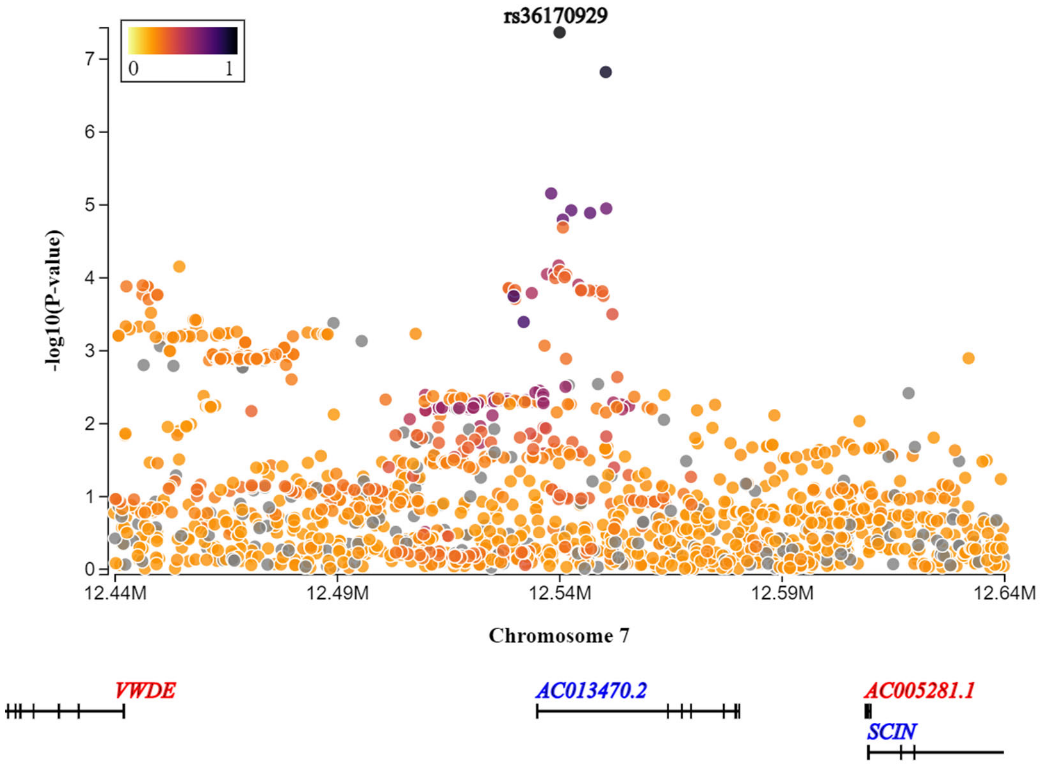

Fig. S3 Regional plots for associations in the regions surrounding rs36170929 according to the results of GWAS analysis

Note: LD-clumped SNPs with rs36170929 located within 1 Mb.

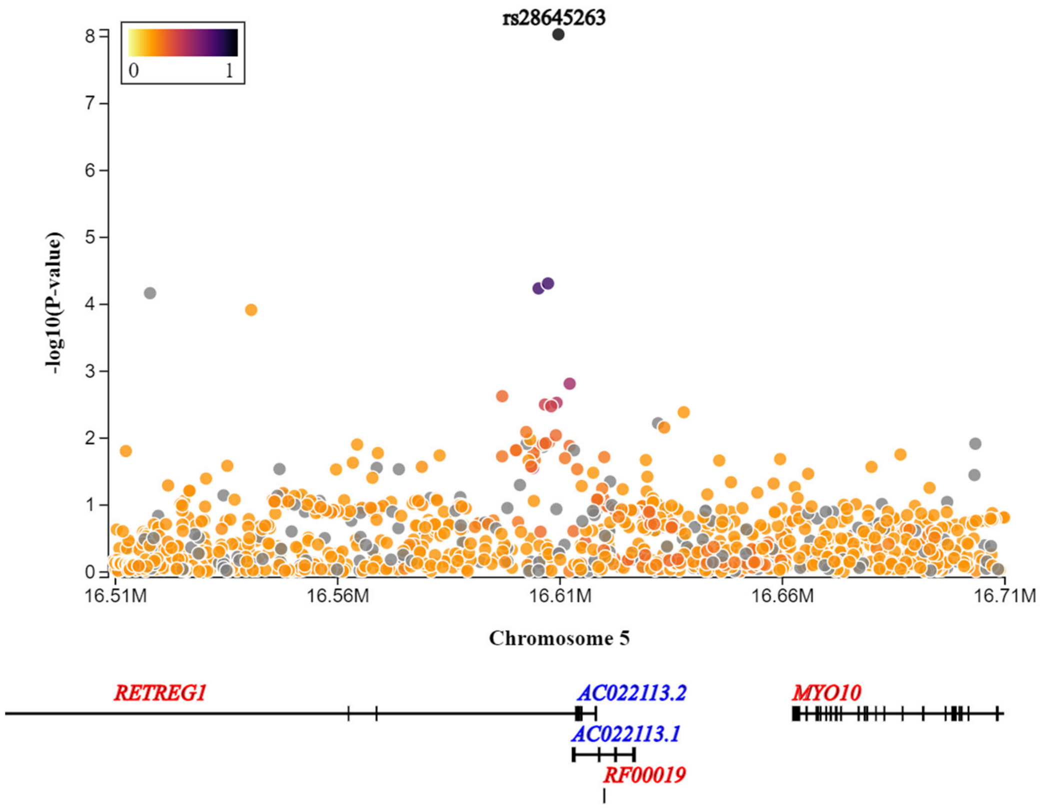

Fig. S4 Regional plots for associations in the regions surrounding rs28645263 according to the results of GWAS analysis

Note: LD-clumped SNPs with rs28645263 located within 1 Mb.

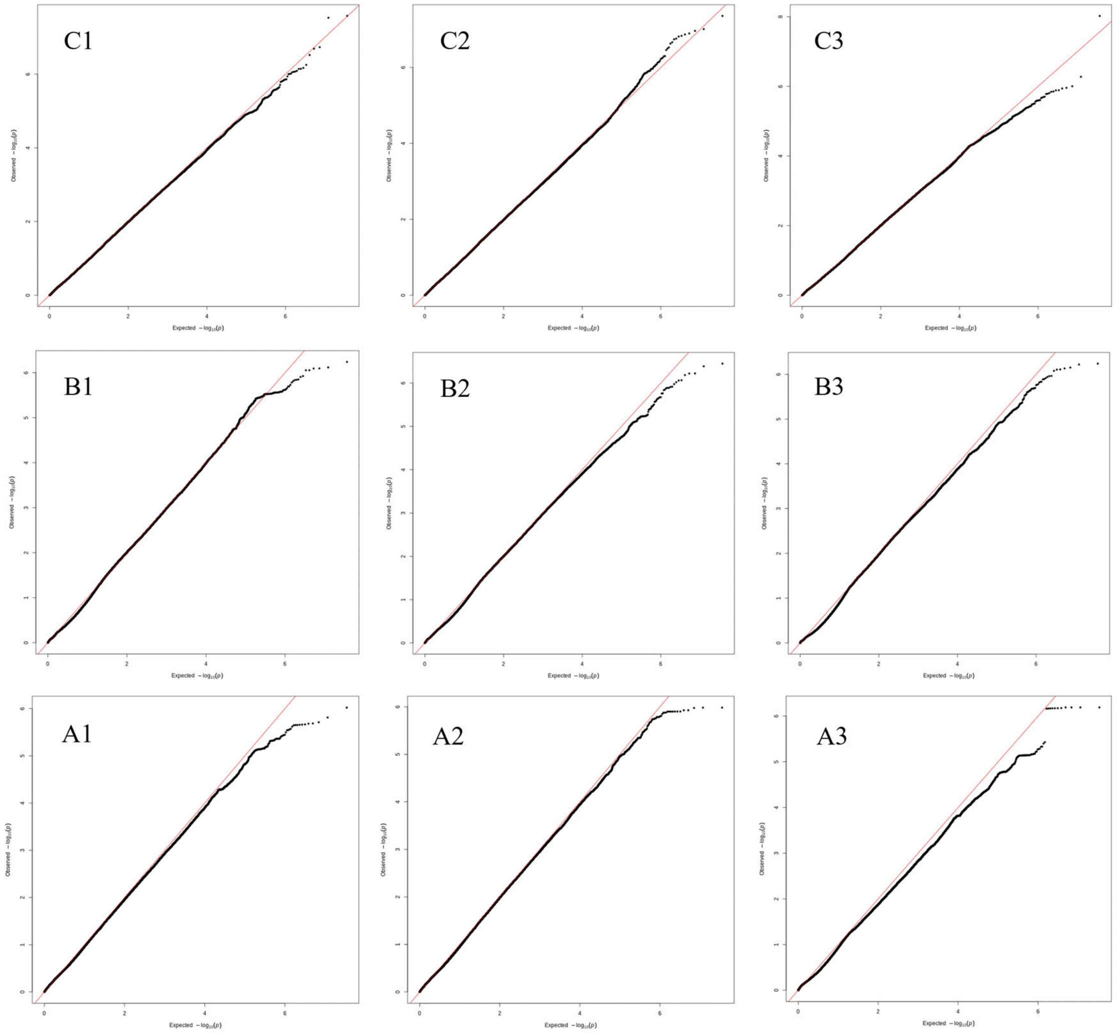

Fig.S5 Quantile-Quantile (QQ) plots for GWAS analyses across study scenarios

Note: The details of the definition of Model A1-C3 are shown in Figure 1b.

Supplementary Text

Supplementary discussion

In our results, different approaches (GWAS, Gene-Based Analysis, and Pathway analysis) did identify different genes. Several reasons may explain the differences. 1) Methodological differences: GWAS identifies individual SNPs with strong association signals, which often correspond to specific loci. In contrast, gene-based analysis aggregates SNP signals across a gene region, potentially detecting cumulative effects of multiple variants with modest effects that may be missed by single-SNP tests. Pathway analysis further aggregates signals across biologically related genes, identifying functional networks and pathways that may be involved in disease susceptibility even if individual genes do not reach genome-wide significance. 2) Biological interpretation: Because genetic susceptibility often involves complex interactions among multiple genes and pathways, these complementary approaches provide different but overlapping perspectives. GWAS highlights specific loci, gene-based tests capture broader gene-level associations, and pathway analysis reveals the collective contribution of genes within

biological pathways. This explains why distinct but biologically related findings can emerge from these analyses.

More discussion regarding the QQ plots and the genomic inflation factor (λ) for each of our main GWAS models

Overall, the λ values ranged from 0.570 to 1.025. The majority of the scenarios exhibited λ values close to 1, indicating appropriate control for population stratification and other potential confounding factors.

For example, in the C2 scenario, the genomic inflation factor was 1.025, suggesting minimal inflation and good calibration of the test statistics. Similarly, values for "C1" ($\lambda = 0.981$), "A2" ($\lambda = 0.974$), and "A1" ($\lambda = 0.983$) also reflect well-controlled analyses. A λ value slightly below 1, as observed in "C3" ($\lambda = 0.943$), "B2" ($\lambda = 0.905$), and "B1" ($\lambda = 0.848$), suggests a conservative test statistic distribution, potentially reducing the risk of false positives. However, these values still fall within acceptable bounds and do not indicate serious deflation. Notably, two scenarios—"A3" ($\lambda = 0.751$) and "B3" ($\lambda = 0.570$)—exhibited substantially lower λ values. This suggests potential deflation of test statistics, which may reduce statistical power and obscure true associations. These conservative results could be due to smaller sample sizes or lower event rates within these subgroups, leading to reduced variation and test statistic shrinkage. Taken together, these λ values suggest that the GWAS analyses were generally well-calibrated, with most scenarios showing minimal inflation or mild deflation.

The QQ plots presented in Figure S5 illustrate the distribution of observed p-values against the expected null distribution for each GWAS scenario:

1) scenarios C1–C3 exhibit slight inflation at the tail, suggesting a modest enrichment of true associations with limited genomic inflation. These plots appear to maintain good control of type I error, with genomic inflation factors (λ) close to 1 (e.g., $\lambda = 0.943$ to 1.025).

2) scenarios B1–B3 show more pronounced deviations from the null line, particularly in B3, indicating potential polygenic signals. However, the λ values (e.g., 0.570 to 0.905) suggest some deflation in test statistics, possibly due to small sample sizes.

3) scenarios A1–A3 similarly display mild deflation (e.g., $\lambda = 0.751$ to 0.983), with QQ plots aligning closely with the null expectation across most p-value ranges.

Overall, the QQ plots show no evidence of substantial inflation across scenarios, supporting the robustness of the analytical pipeline. The slight deviations at the extreme p-values may reflect a few potentially true associations worthy of further investigation.
