# Supplementary figures and images for: Genome-Wide Association Study of COVID-19 Breakthrough Infections and Genetic Overlap with Other Diseases: A Study of the UK Biobank
Source: Int J Mol Sci. 2025 Jul 4;26(13):6441. doi: 10.3390/ijms26136441 (PMC12249495; doi:10.3390/ijms26136441)

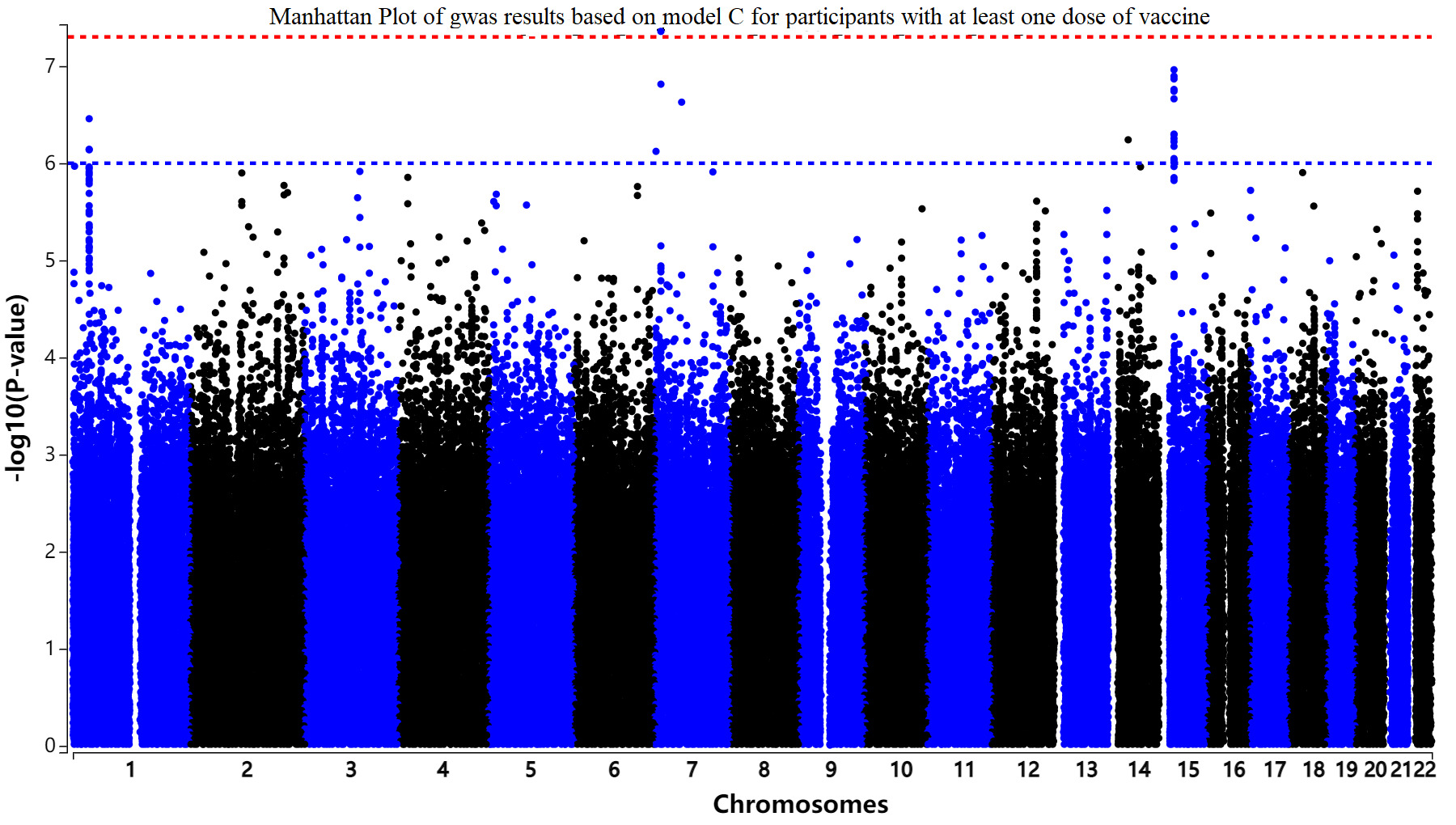

Supplement: Supplementary file 1 [file ijms-26-06441-s001.zip › Figure-S1.jpeg]

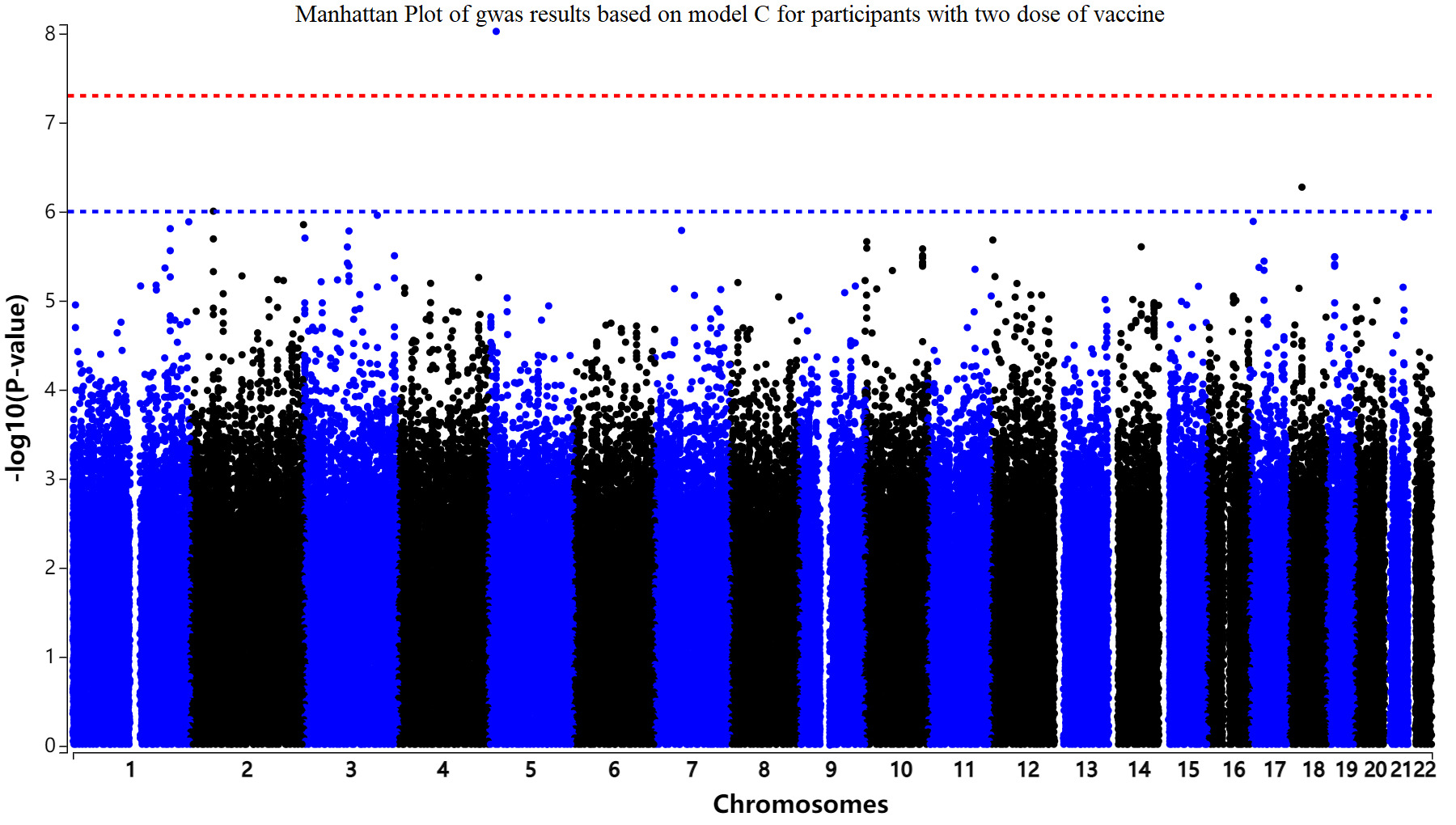

Supplement: Supplementary file 1 [file ijms-26-06441-s001.zip › Figure-S2.jpeg]

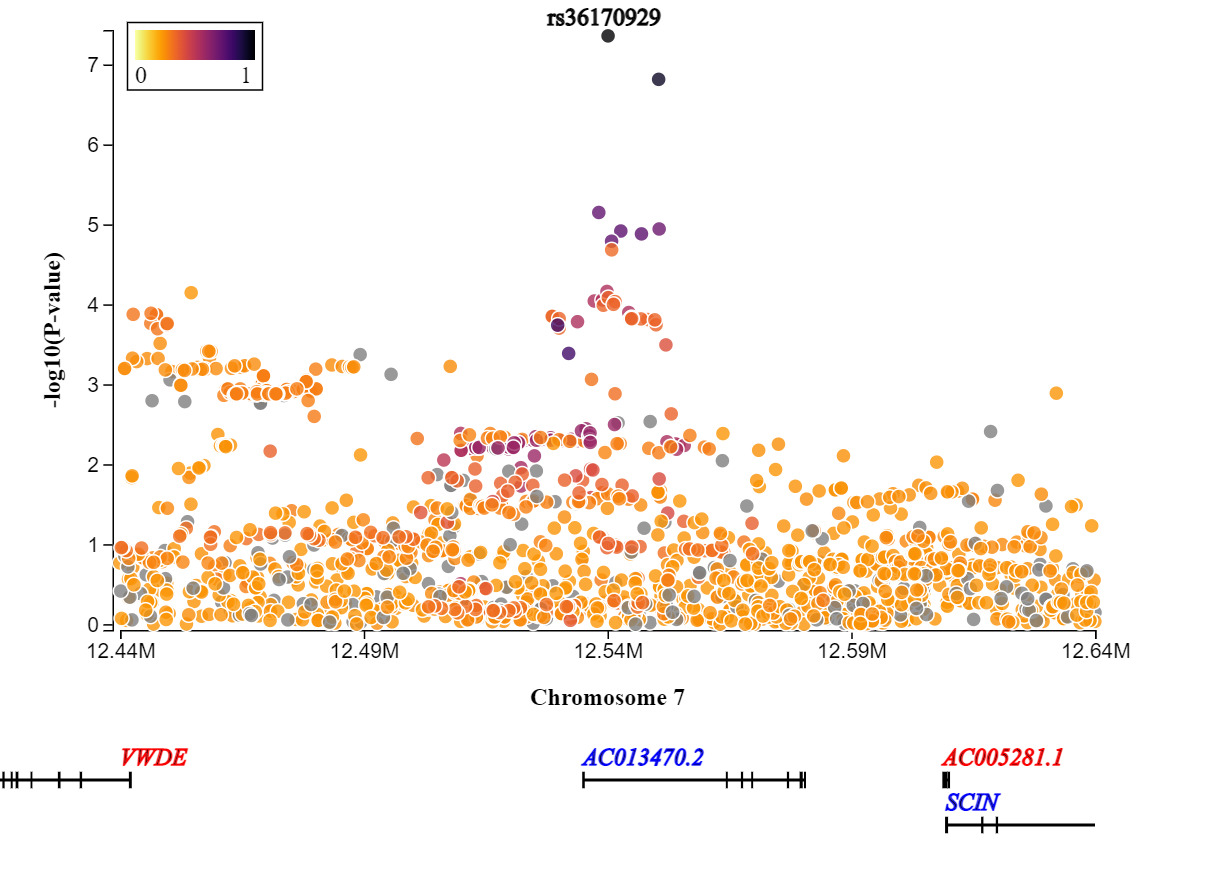

Supplement: Supplementary file 1 [file ijms-26-06441-s001.zip › Figure-S3.jpeg]

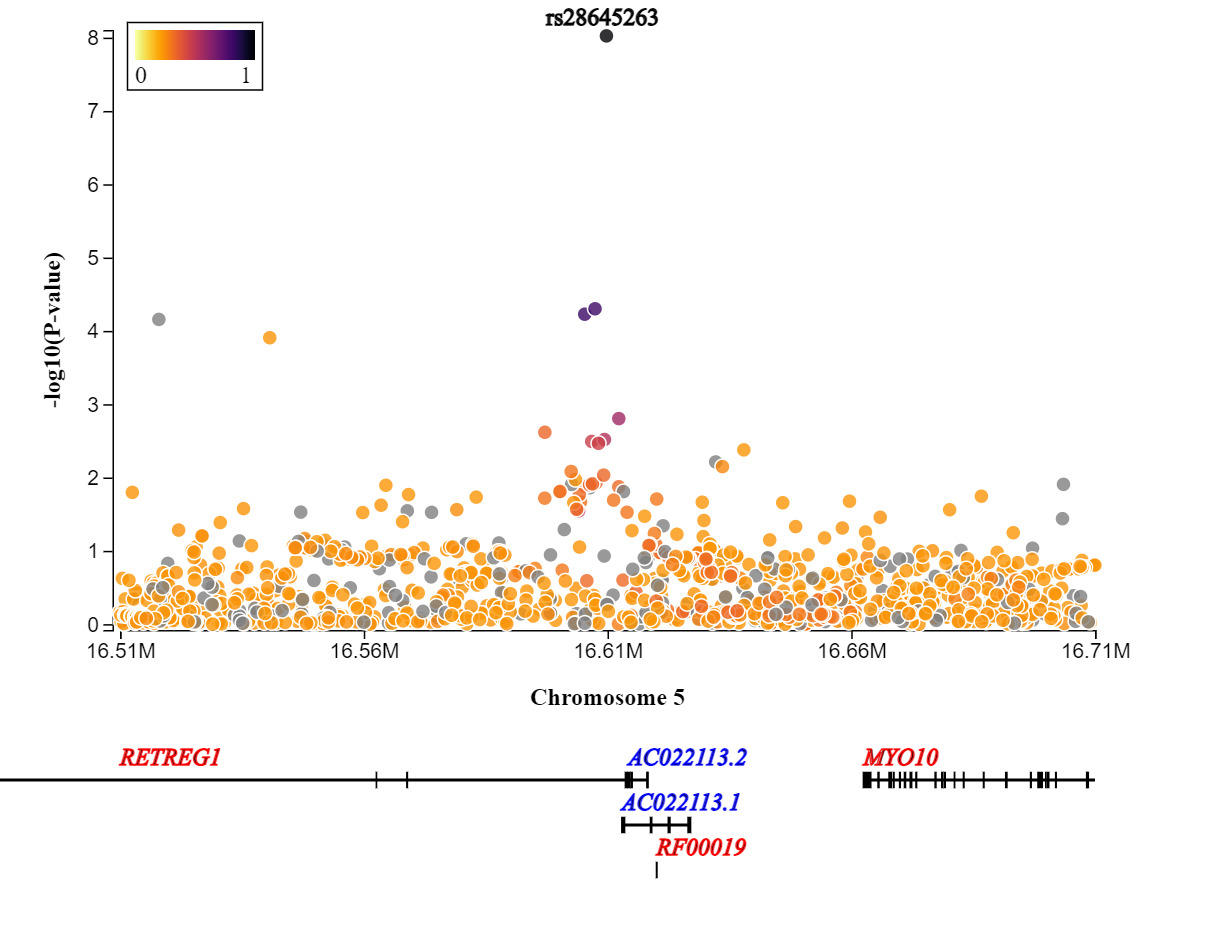

Supplement: Supplementary file 1 [file ijms-26-06441-s001.zip › Figure-S4.jpeg]

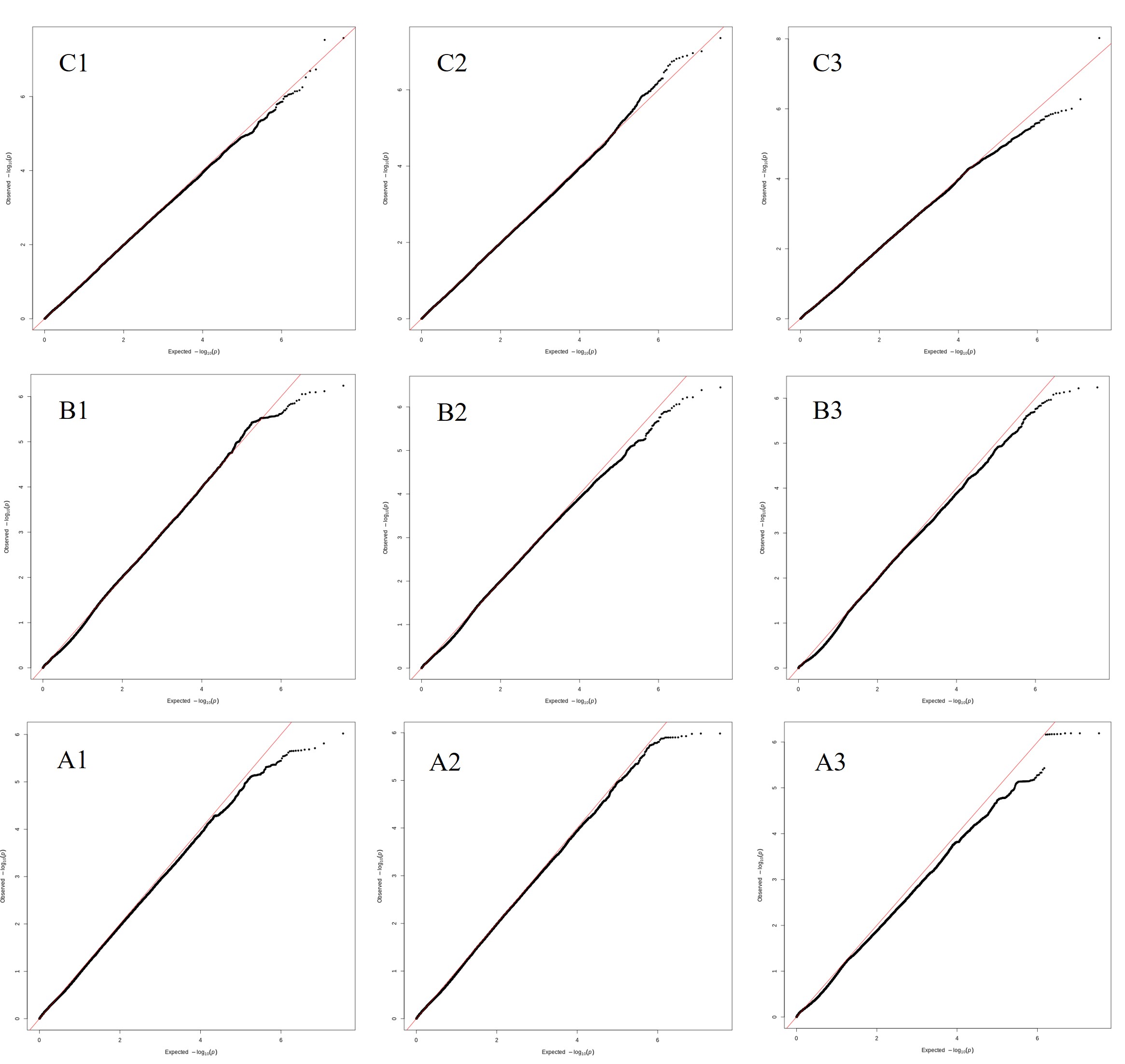

Supplement: Supplementary file 1 [file ijms-26-06441-s001.zip › Figure-S5.jpg]
